# Effects of Semaglutide and Empagliflozin on Inflammatory Markers in Patients with Type 2 Diabetes

**DOI:** 10.3390/ijms24065714

**Published:** 2023-03-16

**Authors:** Ingrid Reppo, Maili Jakobson, Vallo Volke

**Affiliations:** 1 Institute of Biomedicine and Translational Medicine, University of Tartu, 50411 Tartu, Estonia; 2 Endocrinology Unit, Tartu University Hospital, 50406 Tartu, Estonia

**Keywords:** type 2 diabetes, inflammatory markers, semaglutide, empagliflozin

## Abstract

Low-grade inflammation is associated with complications of type 2 diabetes. Glucagon-like peptide-1 receptor agonists and sodium-glucose transporter-2 inhibitors have shown cardioprotective effects that are independent of their glucose-lowering effects. Cardio-protection could be mediated by the anti-inflammatory effects of these medications, but there is currently limited evidence to support this hypothesis. We conducted a prospective clinical study in patients with type 2 diabetes requiring treatment intensification. Ten patients were assigned to receive empagliflozin 10 mg and 10 patients to receive s/c semaglutide (titrated to 1 mg once a week) in a non-randomised manner. All parameters were measured at baseline and after 3 months. Fasting plasma glucose and glycated haemoglobin improved significantly in both treatment groups, with no between-group differences. Body weight and body mass index reduced significantly more in the semaglutide group, whereas waist circumference decreased only in the empagliflozin group. There was a trend for high-sensitivity CRP reduction in both treatment groups that did not reach statistical significance. Interleukin-6 and the neutrophil-to-lymphocyte ratio did not change in either group. Ferritin and uric acid decreased significantly only in the empagliflozin group, and ceruloplasmin decreased significantly only in the semaglutide group. Though there were clinically meaningful improvements in diabetes control in both treatment arms, we could detect only minor changes in some inflammatory markers.

## 1. Introduction

The use of novel classes of diabetes medications, such as glucagon-like peptide-1 receptor agonists (GLP-1 RAs) and sodium-glucose transporter-2 (SGLT-2) inhibitors, has been associated with cardiovascular benefits. The cardioprotective effects of these drugs seem to be independent of their glucose-lowering effects [1,2,3]. Both of the drug classes have multiple auxiliary effects besides glucose control, and the potential mechanism mediating cardiovascular benefit remains elusive. Low-grade inflammation is associated with insulin resistance and hyperglycaemia [4,5,6,7,8] and is a known driver of complications of type 2 diabetes [9]. 

Both of these new drug classes have demonstrated inconsistent anti-inflammatory effects in clinical trials. The SGLT-2 inhibitor canagliflozin has been shown to reduce interleukin-6 (IL-6) levels compared to sulphonyl urea (glimepiride) in patients with type 2 diabetes, but a trend towards a decrease in C-reactive protein (CRP) levels in the canagliflozin group did not reach statistical significance [10]. In another study, empagliflozin and canagliflozin reduced circulating levels of IL-6 in men with type 2 diabetes [11]. Moreover, Iannantuoni et al. showed a significant decrease in high-sensitivity C-reactive protein (hsCRP) levels in type 2 diabetic patients, after a 24-week treatment with empagliflozin [12]. In a trial comparing oral semaglutide (14 mg) and empagliflozin (25 mg) in type 2 diabetic patients only semaglutide reduced CRP [13]. In a weight management trial in non-diabetic obese patients, oral semaglutide reduced hsCRP by 43% compared to placebo, but the statistical significance was lost after adjusting for changes in body weight [14]. Liraglutide has been shown to decrease IL-6 in type 1 diabetic patients [15] and hsCRP in patients with type 2 diabetes [16]. 

Data regarding the possible direct effect of SGLT-2 inhibitors and GLP-1 RAs on immune cells in type 2 diabetes patients are scarce. Borzouei et al. found that empagliflozin showed anti-inflammatory effects by reducing the proliferation of Th cells, decreasing Th17-related factors, and increasing regulatory T cell properties [17]. 

Hence, the anti-inflammatory effect is one of the potential mechanisms to consider in the context of the cardiovascular benefits of novel antidiabetics, but the evidence is still limited. The current study thus aims at directly comparing the effects of s.c. semaglutide and empagliflozin.

## 2. Results

The baseline characteristics of the study participants are given in Table 1 and Supplementary Table S1.

All recruited patients completed the 3-month treatment period with either empagliflozin or semaglutide. On week nine, all patients in the semaglutide group reached the target dose of 1 mg once a week.

### 2.1. Glycaemic Control

**Figure 1:**
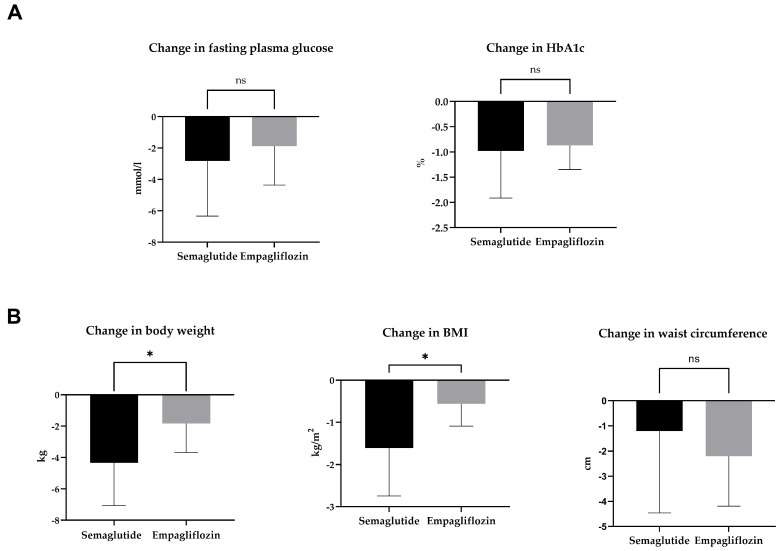
Change in glycaemic control (**A**) and anthropometric parameters (**B**) after 3-month treatment with semaglutide or empagliflozin. Statistics: Unpaired *t*-test; mean ± SD; * *p* < 0.05; ns: *p* ≥ 0.05.

The HbA1c and fasting plasma glucose decreased significantly in both treatment groups. There was no between-group difference in the change in HbA1c or fasting plasma glucose levels (Table 1, Figure 1A).

### 2.2. Body Weight, BMI, and Waist Circumference

The body weight and body mass index (BMI) changed more in the semaglutide group. There was a significant decrease in waist circumference in the empagliflozin group but not in the semaglutide group (Table 1, Figure 1B).

### 2.3. Inflammatory Parameters

**Figure 2:**
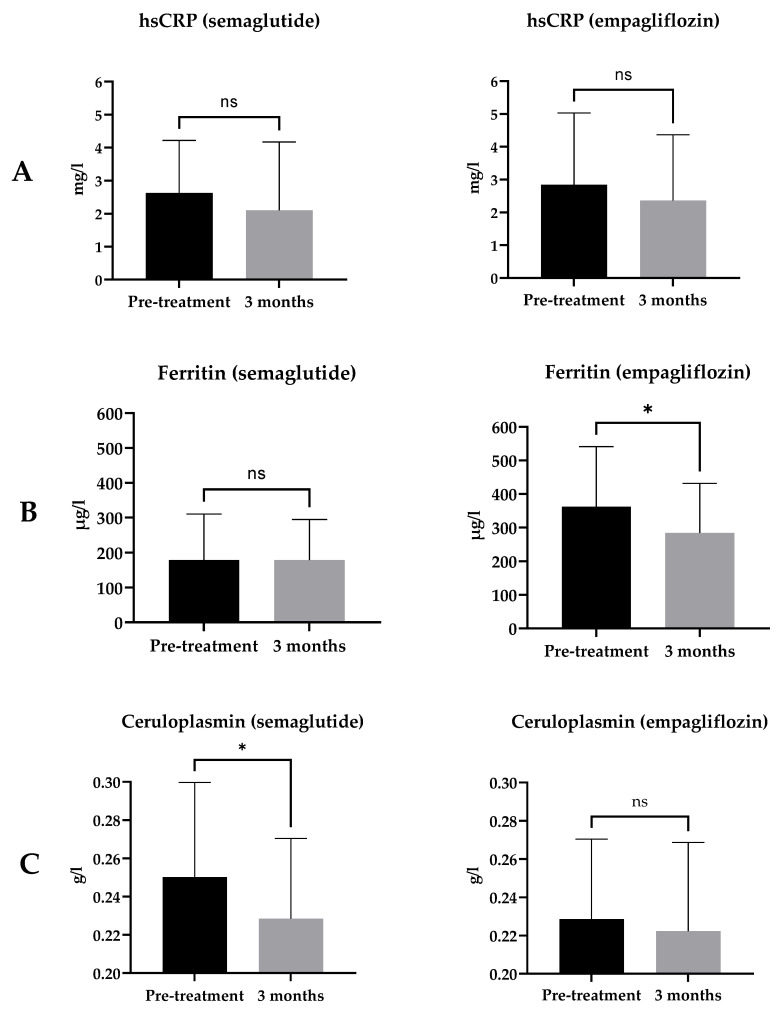
Change in hsCRP (**A**), IL-6 (**B**), ferritin (**C**), and ceruloplasmin (**D**) with 3-month treatment with semaglutide or empagliflozin. Statistics: Paired *t*-test or Wilcoxon matched-pairs signed rank-test as appropriate; mean ± SD; ns: *p* ≥ 0.05, * *p* < 0.05.

The classical inflammatory markers hsCRP and IL-6 did not change during the 3-month treatment period in either treatment group (Table 1, Figure 2A,B). Acute-phase protein ferritin decreased significantly in the empagliflozin group but not in the semaglutide group (Table 1, Figure 2C). Ceruloplasmin, another acute-phase protein produced in the liver that plays a role in copper metabolism and may have a role in the pathogenesis of metabolic diseases such as type 2 diabetes [18], decreased significantly in the semaglutide group but not in the empagliflozin group (Table 1, Figure 2D).

### 2.4. Neutrophil-to-Lymphocyte Ratio

**Figure 3:**
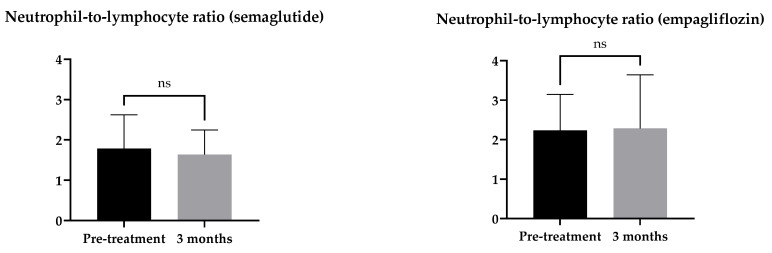
Change in neutrophil-to-lymphocyte ratio with a 3-month treatment with semaglutide or empagliflozin. Statistics: Wilcoxon matched-pairs signed rank-test; mean ± SD; ns *p* ≥ 0.05.

The neutrophil-to-lymphocyte ratio (NLR) is an indicator of inflammation and a predictor of mortality [19], and an increased NLR is positively associated with diabetes complications [20,21,22]. The neutrophil-to-lymphocyte ratio did not change in either treatment group (Figure 3). 

### 2.5. Uric Acid

**Figure 4:**
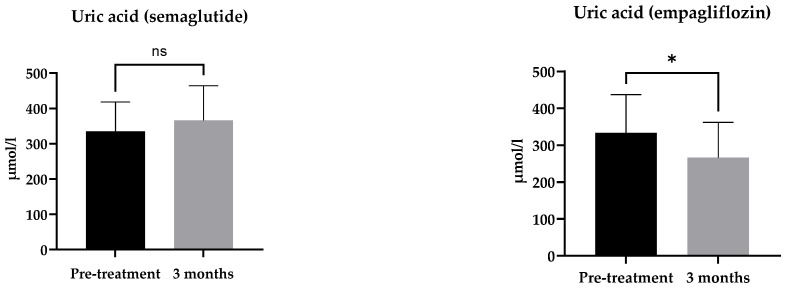
Change in uric acid after a 3-month treatment with semaglutide or empagliflozin. Statistics: paired *t*-test; mean ± SD, ns: *p* ≥ 0.05; * *p* < 0.05.

The uric acid level has been positively related to many inflammatory markers, including CRP [23]. In the semaglutide group, there was no significant change in uric acid level (*p* = 0.256). In the empagliflozin group, there was a significant decrease in the uric acid level (*p* = 0.0104) (Figure 4). 

### 2.6. Glycaemic Control

The HbA1c and fasting plasma glucose decreased significantly in both treatment groups. There was no between-group difference in the change in HbA1c or fasting plasma glucose levels (Table 1, Figure 4A). 

### 2.7. Body Weight, BMI, and Waist Circumference

The body weight and body mass index (BMI) changed more in the semaglutide group. There was a significant decrease in waist circumference in the empagliflozin group but not in the semaglutide group (Table 1, Figure 4B).

### 2.8. Erythrocyte and Iron Metabolism Parameters

The results of red blood cells and iron metabolism parameters are shown in Supplementary Table S2. Haemoglobin and haematocrit increased significantly in the empagliflozin group. Mean corpuscular haemoglobin, iron, soluble transferrin saturation, and transferrin did not change in either treatment group.

## 3. Discussion

The principal finding of our study is that subtle changes in some inflammatory markers occurred during a 3-month treatment with s.c. semaglutide and empagliflozin in this small clinical trial on T2DM patients with suboptimal glycaemic control.

Patients who received s.c. semaglutide had a significantly longer diabetes duration, used fewer statins, and tended to receive more anti-diabetic drugs than patients in the empagliflozin group. Though the differences in background medications did not reach statistical significance, their effect on the study results cannot be excluded.

The 3-month treatment resulted in clinically meaningful reductions in fasting glycaemia, HbA1c, body weight, and BMI in both treatment groups. The improvement in glycaemic control was comparable between the groups; hence, any inter-group differences can be considered independent of the medications’ anti-hyperglycaemic effect. 

Interestingly, the waist circumference decreased significantly in the empagliflozin group but not in the semaglutide group, where the weight and BMI reductions were more prominent. This finding may indicate an initial difference in the pattern of weight loss between the drug classes. However, a longer study has demonstrated a rather similar change in waist circumference with empagliflozin and oral semaglutide after 52 weeks of treatment [13]. 

We could not detect any significant changes in hsCRP with either medication, although there was a trend towards a decrease in hsCRP in both treatment groups. Studies with GLP-1 RAs have consistently shown reductions in hsCRP [13,14,16], but the effect of SGLT-2 inhibitors on hsCRP has been less obvious [10,12]. Ianntuoni et al. reported a significant reduction in hsCRP in 15 type 2 diabetic patients who received empagliflozin 10 mg once a day for 24 weeks [10,12]. On the other hand, Garvey et al. evaluated the effect of canagliflozin and glimepiride on inflammatory biomarkers in a much larger group of type 2 diabetic patients (200 patients in total) and a longer study (52 weeks), which only detected a trend towards a decrease in CRP levels [10]. 

We could not see significant changes in IL-6 in either treatment arm, though IL-6 has decreased in previous studies with SGLT-2 inhibitors [10,11]. Our results are in line with a study showing no effect of liraglutide on IL-6 in type 2 diabetic patients [24]. 

Our study showed clinically significant improvements in fasting plasma glucose and HbA1c in both treatment groups, but contrary to previous studies with type 2 diabetic patients [22,25] where improved glycaemia was associated with NLR reduction, we did not detect any significant changes in the neutrophil-to-lymphocyte ratio in either treatment arm. 

There was a significant decrease in ferritin levels in the empagliflozin group, whereas ferritin levels were stable in semaglutide-treated patients. It is important to point out that the baseline ferritin level was significantly higher in the empagliflozin group vs. the semaglutide group, a drawback of the non-randomised design of the study. This baseline difference can be partially explained by the predominantly male patients in the empagliflozin group, as men have higher ferritin levels [26]. Moreover, six patients in the empagliflozin group had ferritin levels over the upper limit of normal (ULN), while all levels in the semaglutide group were within the reference range. 

Empagliflozin has been shown to decrease ferritin levels in previous studies [27,28]. In our study, there were no changes in other inflammatory markers in the empagliflozin group, and the decrease in ferritin was accompanied by a significant increase in haemoglobin and haematocrit. Hence, we can conclude that these changes probably reflect better iron handling and not an anti-inflammatory effect. This is further supported by the trend towards an increase in sTfR in the empagliflozin group, which reflects increased erythropoietic activity. It would be interesting to see whether SGLT-2 inhibitors may change the membrane fluidity of red blood cells, a potential novel marker of diabetes complications [29,30]. 

In our study, ceruloplasmin levels behaved differently from ferritin. The ceruloplasmin level decreased significantly in the semaglutide group but not in the empagliflozin group. This finding is in line with previous studies by Savchenko et al. [31] and Ekhzaimy et al. [32], which have also demonstrated a decrease in ceruloplasmin levels after treatment with GLP-1 RA liraglutide. Interestingly, the study by Sharma et al. [33] showed that hyperglycaemia correlates positively with ceruloplasmin level and helps to discriminate diabetic patients from non-diabetics. However, we cannot exclude the possible confounding effect of weight change in our study. 

Uric acid decreased significantly after treatment with empagliflozin but not with semaglutide. As other inflammatory markers did not accompany the decrease in uric acid, and SGLT-2 inhibition has been demonstrated to increase the urinary excretion of uric acid [34], this decrease does not seem to reflect a direct anti-inflammatory effect.

To summarise, the treatment effects on biomarkers of inflammation in the current study remained modest, and semaglutide outperformed empagliflozin. The key change was a decrease in ceruloplasmin levels with semaglutide. We could also detect a decrease in hsCRP with both drugs that did not reach statistical significance. It is possible that a larger sample size or longer duration could result in statistical significance. The hsCRP has been considered a novel risk factor for cardiovascular events [35]. However, linking the change in hsCRP directly with the treatment effect of cardioprotective drugs has been challenging. Colchicine is a potent anti-inflammatory drug that prevents cardiovascular events after an acute myocardial infarction. Still, the drug did not change hsCRP or leukocyte counts in these patients [36]. In our study, there were no changes in IL-6 and NLR. Collectively, we were unable to confirm major effects on inflammatory parameters after treatment with s.c. semaglutide or empagliflozin.

There are important limitations to our study. These include the open and non-randomised design, the small sample size, and the uneven sex distribution between treatment groups. Despite the non-randomised design of the study, the recruited patients had similar baseline characteristics regarding anthropometric and biochemical parameters. Due to the post hoc nature of the analyses, there are no prior power calculations for determining an adequate sample size. The list of measured inflammatory markers is not fully comprehensive, as the dataset of inflammatory markers was collected as part of exploratory outcomes and markers not routinely used were not measured. 

The strengths of our study include the prospective design, the use of an active comparator, and the exclusion of patients who were using medications that could affect inflammatory markers. 

We conclude that GLP-1 RAs and SGLT-2 inhibitors reduce the inflammatory markers to some extent, but this effect is not robust enough to explain the cardio-protection seen with these drug classes in type 2 diabetic patients. 

## 4. Materials and Methods

### 4.1. Study Design

We report the results of a prospective pragmatic clinical trial conducted in the Tartu University Hospital endocrinology outpatient clinic in patients with type 2 diabetes.

The trial was designed to study the effects of semaglutide and empagliflozin on the secretion of adrenal hormones and intestinal microbiota composition. The primary endpoint of the trial will be reported elsewhere. The dataset of inflammatory markers reported here was collected as part of exploratory outcomes. 

### 4.2. Inclusion Criteria

The study inclusion criteria were: (1) age ≥ 18 years; (2) type 2 diabetes; (3) HbA1c < 10%; (4) body mass index (BMI) ≥ 32 kg/m^2^; (5) no change in diabetes treatment ≥ 90 days before recruitment to the study; (6) daily metformin dose ≥ 1.5 g; and (7) no prior GLP-1 RA or SGLT-2 inhibitor use.

### 4.3. Exclusion Criteria

The exclusion criteria were: (1) use of oral or intravenous antibiotics ≤ 60 days before recruitment; (2) use of spironolactone ≤ 60 days before recruitment; (3) use of glucocorticoids, cytostatic medications, and biological treatments; (4) pregnancy; (5) use of oral contraceptives or hormone replacement therapy; (6) malignancy; (7) heart failure (NYHA class III-IV); and (8) liver failure.

### 4.4. Study Medications

The decision to start GLP-1 RA semaglutide or SGLT-2 inhibitor empagliflozin was based on clinical judgement of the need for treatment intensification. The patient classification was completed by one investigator (IR).

Semaglutide was started at 0.25 mg (*s*/*c*) once a week. If well tolerated, the dose of semaglutide was increased to 0.5 mg in week five and to 1 mg in week nine. In the empagliflozin group, a 10 mg daily dose was used.

### 4.5. Study Time Points

The pre-specified time points for blood samples and clinical status reported here are baseline and 3 months after recruitment. Weight, height, waist circumference, and blood pressure were also measured at baseline and 3 months after recruitment.

### 4.6. Study Approvals

The trial was approved by the Research Ethics Committee of the University of Tartu (290/T-20) and registered at Clinicaltrials.gov (NCT04151849). The study was conducted following the Declaration of Helsinki. Written informed consent was obtained from all subjects involved in the study.

### 4.7. Laboratory Analyses

Fasting blood samples were obtained between 8 and 10 a.m. All laboratory parameters were measured at the accredited laboratory of Tartu University Hospital using standard methods. A turbidimetric immunoassay was used to measure hsCRP (Cobas 501, Roche Diagnostics GmbH, Germany), ceruloplasmin (Cobas Integra 400 Plus, Roche Diagnostics GmbH, Germany), soluble transferrin receptors (Cobas Integra 400 Plus, Roche Diagnostics GmbH, Germany), transferrin (Cobas 501, Roche Diagnostics GmbH, Germany), and transferrin saturation (Cobas 501, Roche Diagnostics GmbH, Germany). The enzymatic colorimetric method was used to measure uric acid (Cobas 501, Roche Diagnostics GmbH, Germany), and the electrochemiluminescence assay (ECLIA) was used to measure ferritin (Cobas e601, Roche Diagnostics GmbH, Germany) and IL-6 (Cobas e402, Roche Diagnostics GmbH, Germany). The complete blood count was analysed with a Sysmex XN-9000/XN-9100 analyser (Sysmex Corporation, Kobe, Japan). 

### 4.8. Statistical Analyses and Data Presentation

All data were analysed with GraphPad Prism 9.4.1 (GraphPad Software, Inc., Boston, MA, USA) and Microsoft Excel. The D’Agostino-Pearson normality test was used to verify a normal distribution. Normally distributed data were analysed with the paired or unpaired *t*-test as appropriate. The Wilcoxon matched-pairs signed-rank test or Mann–Whitney test was used to test paired and unpaired data that were not normally distributed. Fischer’s exact test was used for categorical variables.

The level of statistical significance was set at *p* < 0.05. Data are presented as mean ± standard deviation (SD).

## 5. Conclusions

The current study compared the effects of 3-month treatment with s.c. semaglutide and empagliflozin on inflammatory markers in patients with type 2 diabetes. Though there were clinically meaningful improvements in diabetes control in both treatment arms, we could detect only minor changes in some inflammatory markers.

## Figures and Tables

**Table 1:** Clinical and biochemical parameters at baseline and after treatment.

	Semaglutide (*n* = 10)			Empagliflozin (*n* = 10)		
Variable	Baseline	3 Months	*p*-Value (Treatment)	Baseline	3 Months	*p*-Value (Treatment)
hsCRP (mg/mL)	2.63 ± 1.59	2.1 ± 2.07	0.2176	2.84 ± 2.19	2.36 ± 2.0	0.3750
IL-6 ^#^	4.52 ±1.92	4.2 ±1.47	0.625	3.80 ±1.90	6.13 ±5.0	0.148
Ferritin (µg/L)	178.9 * ± 131	178.6 ± 116.3	0.9900	362 ± 179	285 ± 146.6	0.0268
HbA1c (%)	8.53 ± 1	7.55 ± 1.13	0.0089	8.13 ± 0.96	7.26 ± 0.74	0.0003
Fasting plasma glucose (mmol/L)	11.9 ± 3.5	9.1 ± 2.5	0.0312	10.7 ± 2.2	8.8 ± 1.6	0.0488
Body weight (kg)	112.5 ± 13.2	108.2 ± 13.9	<0.0007	112.0 ± 17	110.2 ± 16.3	0.0115
BMI (kg/m^2^)	40.2 ± 4.4	38.6 ± 4.1	0.0016	36.6 ± 6.1	36.1 ± 6.2	0.0086
Waist circumference (cm)	123.5 ± 9	122.3 ± 10.5	0.2742	125.1 ± 13.4	122.9 ± 12.8	0.0067

Data are expressed as mean ± SD. * Significant difference in basal values between treatment groups, *p* < 0.05, ^#^ n = 8. hsCRP—high sensitivity C-reactive protein; HbA1c—glucated hemoglobin A1c; BMI—body mass index.

## Data Availability

The data presented in this study are available on reasonable request from the corresponding author. The data are not publicly available due to ethical restrictions.

## Supplementary Materials

The following supporting information can be downloaded at: https://www.mdpi.com/article/10.3390/ijms24065714/s1.

## References

[1] Zinman B., Wanner C., Lachin J.M., Fitchett D., Bluhmki E., Hantel S., Mattheus M., Devins T., Johansen O.E., Woerle H.J. (2015). Empagliflozin, Cardiovascular Outcomes, and Mortality in Type 2 Diabetes. N. Engl. J. Med..

[2] Marso S.P., Bain S.C., Consoli A., Eliaschewitz F.G., Jódar E., Leiter L.A., Lingvay I., Rosenstock J., Seufert J., Warren M.L. (2016). Semaglutide and Cardiovascular Outcomes in Patients with Type 2 Diabetes. N. Engl. J. Med..

[3] Marso S.P., Daniels G.H., Brown-Frandsen K., Kristensen P., Mann J.F.E., Nauck M.A., Nissen S.E., Pocock S., Poulter N.R., Ravn L.S. (2016). Liraglutide and Cardiovascular Outcomes in Type 2 Diabetes. N. Engl. J. Med..

[4] Lontchi-Yimagou E., Sobngwi E., Matsha T.E., Kengne A.P. (2013). Diabetes Mellitus and Inflammation. Curr. Diabetes Rep..

[5] Elimam H., Abdulla A.M., Taha I.M. (2018). Inflammatory markers and control of type 2 diabetes mellitus. Diabetes Metab. Syndr. Clin. Res. Rev..

[6] Prattichizzo F., Giuliani A., Sabbatinelli J., Matacchione G., Ramini D., Bonfigli A.R., Rippo M.R., De Candia P., Procopio A.D., Olivieri F. (2020). Prevalence of residual inflammatory risk and associated clinical variables in patients with type 2 diabetes. Diabetes Obes. Metab..

[7] Muhammad I.F., Borné Y., Hedblad B., Nilsson P.M., Persson M., Engström G. (2016). Acute-phase proteins and incidence of diabetes: A population-based cohort study. Acta Diabetol..

[8] King D.E., Iii A.G.M., Buchanan T.A., Pearson W.S. (2003). C-Reactive Protein and Glycemic Control in Adults with Diabetes. Diabetes Care.

[9] Van Nguyen D., Shaw L.C., Grant M.B. (2012). Inflammation in the pathogenesis of microvascular complications in diabetes. Front. Endocrinol..

[10] Garvey W.T., Van Gaal L., Leiter L.A., Vijapurkar U., List J., Cuddihy R., Ren J., Davies M.J. (2018). Effects of canagliflozin versus glimepiride on adipokines and inflammatory biomarkers in type 2 diabetes. Metabolism.

[11] Tan S.A., Tan L. (2018). Empagliflozin and canagliflozin attenuate inflammatory cytokines interferon-λ, tumor necrosis factor-α, interleukin-6: Possible mechanism of decreasing cardiovascular risk in diabetes mellitus. J. Am. Coll. Cardiol..

[12] Iannantuoni F., de Marañon A.M., Diaz-Morales N., Falcon R., Bañuls C., Abad-Jimenez Z., Victor V.M., Hernandez-Mijares A., Rovira-Llopis S. (2019). The SGLT2 Inhibitor Empagliflozin Ameliorates the Inflammatory Profile in Type 2 Diabetic Patients and Promotes an Antioxidant Response in Leukocytes. J. Clin. Med..

[13] Rodbard H.W., Rosenstock J., Canani L.H., Deerochanawong C., Gumprecht J., Lindberg S., Lingvay I., Søndergaard A.L., Treppendahl M.B., Montanya E. (2019). Oral Semaglutide versus Empagliflozin in Patients with Type 2 Diabetes Uncontrolled on Metformin: The PIONEER 2 Trial. Diabetes Care.

[14] Newsome P., Francque S., Harrison S., Ratziu V., Van Gaal L., Calanna S., Hansen M., Linder M., Sanyal A. (2019). Effect of semaglutide on liver enzymes and markers of inflammation in subjects with type 2 diabetes and/or obesity. Aliment. Pharmacol. Ther..

[15] Brock C., Hansen C.S., Karmisholt J., Møller H.J., Juhl A., Farmer A.D., Drewes A., Riahi S., Lervang H.H., Jakobsen P.E. (2019). Liraglutide treatment reduced interleukin-6 in adults with type 1 diabetes but did not improve established autonomic or polyneuropathy. Br. J. Clin. Pharmacol..

[16] Anholm C., Kumarathurai P., Pedersen L.R., Samkani A., Walzem R.L., Nielsen O.W., Kristiansen O.P., Fenger M., Madsbad S., Sajadieh A. (2019). Liraglutide in combination with metformin may improve the atherogenic lipid profile and decrease C-reactive protein level in statin treated obese patients with coronary artery disease and newly diagnosed type 2 diabetes: A randomized trial. Atherosclerosis.

[17] Borzouei S., Moghimi H., Zamani A., Behzad M. (2021). Changes in T helper cell-related factors in patients with type 2 diabetes melli-tus after empagliflozin therapy. Hum. Immunol..

[18] Cunninghamn J., Leffell M., Mearkle P., Harmatz P. (1995). Elevated plasma ceruloplasmin in insulin-dependent diabetes mellitus: Evidence for increased oxidative stress as a variable complication. Metabolism.

[19] Song M., Graubard B.I., Rabkin C.S., Engels E.A. (2021). Neutrophil-to-lymphocyte ratio and mortality in the United States general population. Sci. Rep..

[20] Verdoia M., Schaffer A., Barbieri L., Aimaretti G., Marino P., Sinigaglia F., Suryapranata H., De Luca G., Novara Atherosclerosis Study Group (2015). Impact of diabetes on neutrophil-to-lymphocyte ratio and its relationship to coronary artery disease. Diabetes Metab..

[21] Wan H., Wang Y., Fang S., Chen Y., Zhang W., Xia F., Wang N., Lu Y. (2020). Associations between the Neutrophil-to-Lymphocyte Ratio and Diabetic Complications in Adults with Diabetes: A Cross-Sectional Study. J. Diabetes Res..

[22] Verma S., Husain M., Madsen C., Leiter L.A., Rajan S., Vilsboll T., Rasmussen S., Libby P. (2021). Neutrophil-to-lymphocyte ratio predicts cardiovascular events in patients with type 2 diabetes: Post hoc analysis of SUSTAIN 6 and PIONEER 6. Eur. Heart J..

[23] Ruggiero C., Cherubini A., Ble A., Bos A.J., Maggio M., Dixit V.D., Lauretani F., Bandinelli S., Senin U., Ferrucci L. (2006). Uric acid and inflammatory markers. Eur. Hear. J..

[24] Courrèges J.-P., Vilsbøll T., Zdravkovic M., Le-Thi T., Krarup T., Schmitz O., Verhoeven R., Bugáñová I., Madsbad S. (2008). Beneficial effects of once-daily liraglutide, a human glucagon-like peptide-1 analogue, on cardiovascular risk biomarkers in patients with Type 2 diabetes. Diabet. Med..

[25] Duman T.T., Aktas G., Atak B.M., Kocak M.Z., Erkus E., Savli H. (2019). Neutrophil to lymphocyte ratio as an indicative of diabetic control level in type 2 diabetes mellitus. Afr. Health Sci..

[26] Custer E.M., Finch C.A., Sobel R.E., Zettner A. (1995). Population norms for serum ferritin. J. Lab. Clin. Med..

[27] Ferrannini E., Murthy A.C., Lee Y.-H., Muscelli E., Weiss S., Ostroff R.M., Sattar N., Williams S.A., Ganz P. (2020). Mechanisms of Sodium–Glucose Cotransporter 2 Inhibition: Insights from Large-Scale Proteomics. Diabetes Care.

[28] Mazer C.D., Hare G.M., Connelly P.W., Gilbert R.E., Shehata N., Quan A., Teoh H., Leiter L.A., Zinman B., Jüni P. (2020). Effect of Empagliflozin on Erythropoietin Levels, Iron Stores, and Red Blood Cell Morphology in Patients With Type 2 Diabetes Mellitus and Coronary Artery Disease. Circulation.

[29] Bianchetti G., Rizzo G.E., Serantoni C., Abeltino A., Rizzi A., Tartaglione L., Caputo S., Flex A., De Spirito M., Pitocco D. (2022). Spatial Reorganization of Liquid Crystalline Domains of Red Blood Cells in Type 2 Diabetic Patients with Peripheral Artery Disease. Int. J. Mol. Sci..

[30] Bianchetti G., Di Giacinto F., Pitocco D., Rizzi A., Rizzo G.E., De Leva F., Flex A., di Stasio E., Ciasca G., De Spirito M. (2019). Red blood cells membrane micropolarity as a novel diagnostic indicator of type 1 and type 2 diabetes. Anal. Chim. Acta X.

[31] Savchenko L.G., Digtiar N.I., Selikhova L.G., Kaidasheva E.I., Shlykova O.A., Vesnina L.E., Kaidashev I. (2019). Liraglutide exerts an anti-inflammatory action in obese patients with type 2 diabetes. Rom. J. Intern. Med..

[32] Ekhzaimy A.A., Masood A., Benabdelkamel H., Elhassan T., Musambil M., Alfadda A.A. (2022). Plasma proteomics reveals an improved cardio-metabolic profile in patients with type 2 diabetes post-liraglutide treatment. Diabetes Vasc. Dis. Res..

[33] Sharma V.K., Tumbapo A., Pant V., Aryal B., Shrestha S., Yadav B.K., Tuladhar E.T., Bhattarai A., Raut M. (2018). Ceruloplasmin, a potential marker for glycemic status and its relationship with lipid profile in Type II diabetes mellitus. Asian J. Med Sci..

[34] Chino Y., Samukawa Y., Sakai S., Nakai Y., Yamaguchi J., Nakanishi T., Tamai I. (2014). SGLT2 inhibitor lowers serum uric acid through alteration of uric acid transport activity in renal tubule by increased glycosuria. Biopharm. Drug Dispos..

[35] Sharif S., Van der Graaf Y., Cramer M.J., Kapelle L.J., de Borst G.J., Visseren F.L.J., Westerink J., van Petersen R., Dinther B.G.F., Algra A. (2021). Low-grade inflammation as a risk factor for cardiovascular events and all-cause mortality in patients with type 2 diabetes. Cardiovasc. Diabetol..

[36] Tardif J.-C., Kouz S., Waters D.D., Bertrand O.F., Diaz R., Maggioni A.P., Pinto F.J., Ibrahim R., Gamra H., Kiwan G.S. (2019). Efficacy and Safety of Low-Dose Colchicine after Myocardial Infarction. N. Engl. J. Med..

