# Supplementary material for: Effects of Semaglutide and Empagliflozin on Inflammatory Markers in Patients with Type 2 Diabetes
Source: Int J Mol Sci. 2023 Mar 16;24(6):5714. doi: 10.3390/ijms24065714 (PMC10054691; doi:10.3390/ijms24065714)
Supplement: Supplementary file 1 [file ijms-24-05714-s001.zip › ijms-2144434-supplementary.pdf]

Supplementary Materials

Supplementary Table S1. Baseline characteristics of the cohort.

Variable	Semaglutide (n=10)	Empagliflozin (n=10)	p-value
Transferrin saturation (%)	28.6 ±5.8	25.3 ± 5.7	0.212
Soluble transferrin receptors (mg/l)	2.82 ±0.69	2.81 ±0.75	0.976
Iron (µmol/l)	19.8 ±3.6	16.6 ±3.6	0.060
Hemoglobin (g/l)	144 ±13.9	148 ±4.4	0.396
Hematocrit (%)	42.8 ±3.9	44.3 ±1.3	0.266
MCHC (g/l)	337 ±9.2	334 ±5.5	0.435
Creatinine (µmol/l)	76.5 ±30.5	78.2 ±16.2	0.592
Uric acid (µmol/l)	334.9 ±83.2	333.4 ±103.9	0.972

Data are expressed as mean ±SD. Abbreviations: MCHC- *mean corpuscular hemoglobin concentration*.

Supplementary Table S2. Erythrocyte and iron metabolism parameters.

	**Semaglutide**			**Empagliflozin**		
	Pre-treatment	3 months	p-value	Pre-treatment	3 months	p-value
Haemoglobin (g/l)	144 (13.87)	144 (11.5)	0.9708	148	155	<0.0001
Haematocrit (%)	42.8 (3.9)	43.1 (3.1)	0.7606	44.3 (1.3)	46.7 (1.6)	<0.0001
MCHC (g/l)	336.8 (9.2)	334.1 (5.5)	0.4598	334.1 (5.5)	332.9 (9.3)	0.5652
Iron (µmol/l)	19.79 (3.61)	17.72 (4.15)	0.2292	16.57 (3.56)	17.67 (6.84)	0.6421
Soluble transferrin receptors (mg/l)	2.82 (0.69)	2.53 (0.59)	0.0494	2.81 (0.75)	3.08 (0.91)	0.1327
Transferrin saturation (%)	25.57 (9.31)	26.32 (8.38)	0.8274	25.28 (5.66)	25.71 (9.94)	0.8929

Data presented as mean ± SD. Statistics: paired t-test or Wilcoxon matched-pairs signed rank test as appropriate.
